# Atoh1 expression levels define the fate of rat cochlear nonsensory epithelial cells *in vitro*

**DOI:** 10.3892/mmr.2014.2202

**Published:** 2014-04-30

**Authors:** WEN-WEI LUO, JUAN-MEI YANG, ZHAO HAN, YA-SHENG YUAN, HAI-BIN SHENG, XIANG LIU, FANG-LU CHI

**Affiliations:** Department of Otolaryngology-Head and Neck Surgery, Eye and ENT Hospital of Fudan University, Xuhui, Shanghai 200031, P.R. China

**Keywords:** Atoh1 expression level, ectopic hair cell-like cells, lesser epithelial ridge, virus titer

## Abstract

Atonal homolog 1 (Atoh1) is a basic helix-loop-helix transcription factor that is essential for inner ear hair cell differentiation. Previous studies have reported that Atoh1 gene transfer induces the production of ectopic hair cell-like cells (EHCLCs). In the present study, the effect of different Atoh1 expression levels and the duration of EHCLC formation on the lesser epithelial ridge (LER) of cochleae was examined using a human adenovirus serotype 5 (Ad5) vector encoding *atoh1* and the reporter gene *EGFP*. Different *Ad5*-*EGFP*-*atoh1*/*Ad5-EGFP* virus titers were added to cultured cochlear explants and EHCLCs were detected in the LER at various time points. The results demonstrated that GFP alone did not induce EHCLCs. By contrast, Atoh1 expression induced EHCLCs as early as 2.5–5 days following *EGFP*-*atoh1* infection in the LER and depending upon the viral titer, the number of EHCLCs increased with time. Higher *Ad5-EGFP*-*atoh1* titers induced enhanced Atoh1 expression, resulting in an increase in EHCLCs. Lower *Ad5-EGFP-atoh1* titers required more time for EHCLC formation and very low titers of *Ad5*-*EGFP*-*atoh1* induced only weak Atoh1 expression and did not trigger EHCLC formation. In conclusion, the present study utilized an appropriate *Ad5*-*EGFP*-*atoh1* titer range to induce Atoh1 expression and the subsequent production of EHCLCs. The results revealed that the Atoh1 expression level defined the fate of LER cells as either EHCLCs or nonsensory epithelial cells. This evidence may provide an important guideline for future studies into gene therapy strategies for the treatment of deafness.

## Introduction

Hair cells (HCs) transform sound and balance signals into electrical impulses in the cochlear and vestibular end organs. By contrast to vertebrates that are able to spontaneously regenerate new hair and supporting cells (1,2), there is no effective way to stimulate their regeneration in mammals once hair cells have been damaged by noise, ototoxic drugs or aging, which hampers the treatment of sensorin, a neural hearing impairment that is caused by hair cell loss.

Atoh1 is a basic helix-loop-helix transcription factor that is crucial in hair cell formation (3,4). Knockout of *atoh1* in mice results in the absence of differentiated hair cells and supporting cells, while Atoh1 overexpression in cultured explants or *in vivo* induces ectopic hair cell-like cell (EHCLC) formation (3,5–14).

Studies in a novel *atoh1* ‘self-terminating’ mouse model have suggested that Atoh1 expression level and duration is crucial for inner and outer hair cell differentiation *in vivo* (15). Therefore, we aimed to investigate how Atoh1 affects EHCLC formation and whether Atoh1 expression defines the fate of LER cells as either ectopic, newly formed hair cells or nonsensory epithelial cells. In the present study, cultured explants were infected with several virus titers and EHCLC expression was detected in the LER at different time points. It was identified that the formation of EHCLCs was Atoh1 dependent, as no EHCLCs formed upon infection by GFP alone. Following LER infection with an appropriate titer (*EGFP*-*atoh1*) for Atoh1 expression (1.6×10^9^ PFU/ml), EHCLC production was detected as early as 2.5 days and the number of EHCLCs increased with time. Higher *Ad5*-*EGFP*-*atoh1* titers induced increased Atoh1 expression and a larger quantity of hair cell-like cells appeared at earlier time points compared with lower titers. Lower *Ad5*-*EGFP*-*atoh1* titers induced less Atoh1 expression and required a greater duration for EHCLC formation. Extremely low *Ad5*-*EGFP*-*atoh1* titers induced only weak Atoh1 expression and no formation of EHCLCs. Therefore, Atoh1 expression levels define the fate of LER cells as either EHCLCs or nonsensory epithelial cells, and greater Atoh1 expression decreases the time required for EHCLC formation in the LER. These data define an appropriate *Ad5*-*EGFP*-*atoh1* titer range for ectopic hair cell formation and which will act as an important guideline for future studies.

## Materials and methods

### Cultures of postnatal rat cochleae and atoh1 gene infection

This study was approved by the Institutional Animal Care and Animal Ethics Committee of Fudan University (Xuhui, Shanghai, China). One-day-old postnatal (P1) SD rats were used for the experiments and were purchased from Slaccas Experimental Animal Company (Xuhui, Shanghai, China). The rats were sacrificed by CO_2_ asphyxiation. The cochlear explants culture was prepared as described previously (11,12). The final concentrations of the *Ad5*-*EGFP*-*atoh1* vector were 0.1×10^8^, 0.4×10^8^, 0.8×10^8^, 1.6×10^8^ and 2.4×10^8^ PFU/ml in serum-free DMEM/F12. The control group (*Ad5-EGFP*) included corresponding titers. The viruses used were as described previously (6,7,11,12).

### Tissue preparation and immunofluorescence

The cochlear explants were fixed with 4% paraformaldehyde for 30 min and then treated with 0.1% Triton X-100 plus 10% donkey serum for 30 min. Following this, the explants were incubated with the following primary antibodies for 24 h at 4°C; myosin7A (1:100; Proteus Biosciences Inc., Ramona, CA, USA), myosin7A (1:200; Developmental Studies Hybridoma Bank, Iowa City, IA, USA), p27kip1 (1:100; Cell Signaling Technology, Inc., CA, USA) and Sox2 (1:300, Santa Cruz Biotechnology, Inc, Santa Cruz, CA, USA). The preparation was washed 3–5 times in PBS and then incubated with secondary antibodies for 2 h at 37°C in the dark. The secondary antibodies included donkey anti-mouse/rabbit Alexa Fluor 555 (1:1,000) and/or donkey anti-mouse/rabbit/goat (H+L) Alexa Fluor 647 (1:1,000; Molecular Probes, Invitrogen Life Technologies, Carlsbad, CA, USA). The specimens were visualized with a Zeiss LSM 510 confocal laser-scanning microscope (Carl Zeiss, Oberkochen, Germany) and only one image was captured by the microscope.

### Cell counting and statistical analysis

Only cells at the LER region of the mid-basal turns were counted. Using random samples, the cells in 200 μm segments along the length of the cochlea were counted. Each group had at least five different cochlear explants and each explant was sampled at five areas. Ectopic hair cells were counted 3, 5, 7, 9 and 11 days post-infection. All the cell count was precisely performed by manually analyzing the confocal images. The values are expressed as the mean ± standard error and using a one-way ANOVA statistical test when appropriate. P<0.05 was considered to indicate a statistically significant difference.

## Results

### Ad5 vector transfection efficiency in the LER

**Figure 1:**
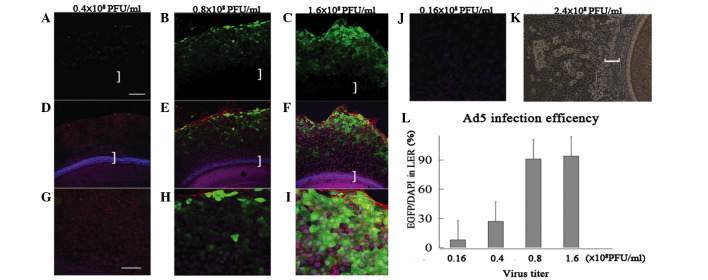
Different Ad5 vector infection rates in the LER at different titers. (A and D) At 0.4×10^8^ PFU/ml, a number of LER cells were GFP positive with moderate green fluorescence. (B and E) At 0.8×10^8^ PFU/ml, the majority of LER cells were GFP positive with moderate to strong green fluorescence. (C and F) Following infection at 1.6×10^8^ PFU/ml, the majority of LER cells were GFP positive with strong green fluorescence. (G–I) Magnified views of (D), (E) and (F). (J) Following infection at 0.16×10^8^ PFU/ml, sporadic cells were GFP positive with weak green fluorescence. (K) Following infection at 2.4×10^8^ PFU/ml, the virus damaged the cochlear explants. (L) Histogram of the *Ad5-EGFP-atoh1/Ad5-EGFP* infection rate (x-axis, virus titer; y-axis, number of GFP/number of DAPI). Green, GFP; blue, myosin7A; purple, DAPI. Scale: A–F, 100 μm; G–J, 50 μm. White brackets indicate the sensory epithelium. LER, lesser epithelial ridge; Ad5, human adenovirus serotype 5.

The *Ad5-EGFP*/*Ad5*-*EGFP*-*atoh1* transfection efficiency in the LER (outside of the outer hair cells) was determined by infection with different virus titers (Fig. 1). At a titer of 0.16×10^8^ PFU/ml, only 8±2% of LER cells were GFP positive with weak green fluorescence (Fig. 1J). At a titer of 0.4×10^8^ PFU/ml, 27±4% of LER cells were GFP positive with moderate green fluorescence (Fig. 1A and 1D and 1G). At 0.8×10^8^ PFU/ml, 91±7% of LER cells were GFP positive with moderate-to-strong green fluorescence (Fig. 1B and 1E and 1H). At 1.6×10^8^ PFU/ml, 94±9% of LER cells were GFP positive with strong green fluorescence (Fig. 1C and 1F and 1I). However, when 2.4×10^8^ PFU/ml was used, the cultured explants disintegrated (Fig. 1K). Higher viral infection efficiency was observed with increasing titer, because the transfection efficiency of 0.4×10^8^ PFU/ml was significantly higher than that of 0.16×10^8^ PFU/ml (n=5, P<0.05) and that of 0.8×10^8^ PFU/ml was significantly higher than that of 0.4×10^8^ PFU/ml (n=5, P<0.05), whereas the transfection efficiency of 1.6×10^8^ PFU/ml was similar to 0.8×10^8^ PFU/ml (n=5, P>0.05). However, the fluorescence intensity at 1.6×10^8^ PFU/ml was higher than at 0.8×10^8^ PFU/ml. Therefore, it was concluded that the most effective virus titer was 1.6×10^8^ PFU/ml.

### Formation of new EHCLCs at the LER is Atoh1 dependent

**Figure 2:**
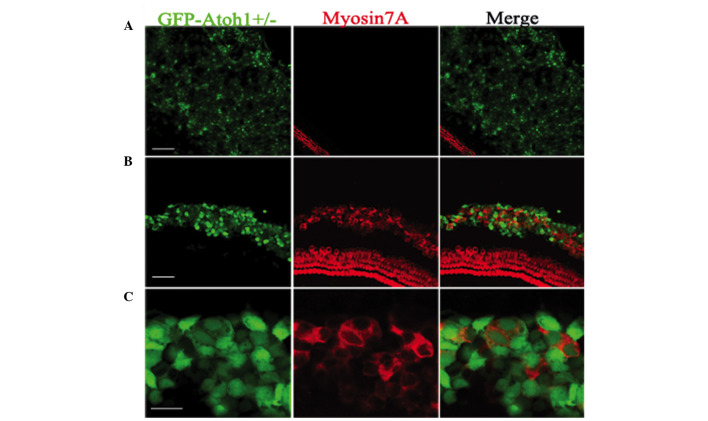
The formation of new EHCLCs at the LER is Atoh1 dependent. Cultured cochlear explants five days following *Ad5-EGFP/Ad5-EGFP-atoh1* infection. (A) *Ad5-EGFP* (green: GFP) did not induce myosin7A (red)-positive cells in the LER. (B) Ad5-EGFP-atoh1 (green: GFP-Atoh1) induced numerous myosin7A (red)-positive cells in the LER. (C) Magnified images from (B). Scale bar: 100 μm in (A) and (B); 50 μm in (C). EHCLCs, ectopic hair-cell-like cells; LER, lesser epithelial ridge; Atoh1, Atonal homolog1; Ad5, human adenovirus serotype 5.

Following *Ad-EGFP* infection of the cultured explants (Fig. 2A), the LER cells presented robust EGFP fluorescence, however no myosin7A-positive cells were observed. The LER cells were unable to differentiate into hair cells. Following *Ad5*-*EGFP*-*atoh1* infection, the LER was the target (Fig. 1B). Consistent with previous studies (11,14), *Ad5*-*EGFP*-*atoh1* infection resulted in the induction of myosin7A-positive cells in the LER regions (Fig. 2B), suggesting that these newly formed hair cells were Atoh1 overexpression dependent. Many of the EGFP-positive cells were myosin7A negative, despite having been infected with *Ad5*-*EGFP*-*atoh1*. To determine whether the Atoh1 expression level or duration led to this phenomenon, different *Ad5*-*EGFP*-*atoh1* titers were utilized, and the quantities and percentages of new hair cells were detected at different time points in the following study.

### EHCLC formation requires a certain Atoh1 expression level

**Figure 3:**
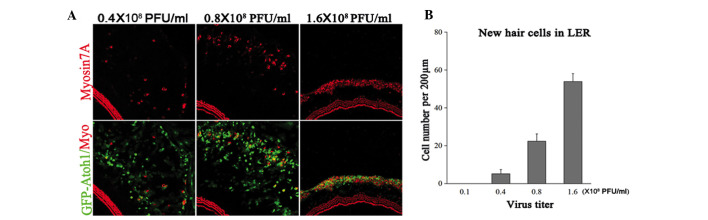
Higher Atoh1 expression levels induced the formation of more EHCLCs in the LER 5 DVI following *Ad-EGFP-atoh1* infection. (A) Increasing the viral titer led to higher Atoh1 expression (GFP: green) and induced more myosin7a-positive (red) cells in the LER. (B) A bar graph illustrates the correlation between the virus titer and the number of ectopic hair cells. The cells in 200 μm segments along the length of the cochlea were counted. Scale bar: 100 μm in (A). Atoh1, Atonal homolog1; EHCLCs, ectopic hair-cell-like cells; LER, lesser epithelial ridge; Ad5, human adenovirus serotype 5; DVI, days after viral infection.

To address whether new EHCLC formation depends on Atoh1 expression, cultured explants were treated with *Ad*-*EGFP*-*atoh1* at four different titers: 0.16×10^8^, 0.4×10^8^, 0.8×10^8^ and 1.6×10^8^ PFU/ml. The samples were fixed at five days following viral infection (DVI) and the numbers of EGFP-myosin7A double-positive cells and EGFP-positive cells were counted in the LER (Fig. 3). At 0.16×10^8^ PFU/ml *Ad5*-*EGFP*-*atoh1*, no myosin7A-positive cells were detected, implying that low Atoh1 expression was unable to induce hair-cell-like cell formation (n=5). At 0.4×10^8^ PFU/ml *Ad5*-*EGFP*-*atoh1*, there were 5±2 myosin7A-EGFP double-positive cells per 200 μm in the LER (4±3% of all EGFP-positive cells). At 0.8×10^8^ PFU/ml *Ad5*-*EGFP*-*atoh1*, there were 22±4 myosin7A-EGFP double-positive cells per 200 μm in the LER (14±5% of all EGFP-positive cells). At 1.6×10^8^ PFU/ml *Ad5*-*EGFP*-*atoh1*, there were 54±4 myosin7A-EGFP double-positive cells per 200 μm in LER (57±13% of all EGFP-positive cells; n=5). The number of EHCLCs in the higher virus titer groups was significantly greater compared with the lower virus titer groups (Fig. 2B). Furthermore, the LER to hair cell-like cell conversion rate was significantly enhanced in the higher than in the lower virus titer group. These data demonstrate that increasing the virus titer increased Atoh1 expression and this subsequently increased the myosin7A-positive cell number in the LER. If Atoh1 expression was too low, few LER cells converted to hair-cell-like cells. Thus, EHCLCs production was dependent on specific Atoh1 expression levels.

### Higher Atoh1 expression reduces the duration of EHCLC formation in the LER

**Figure 4:**
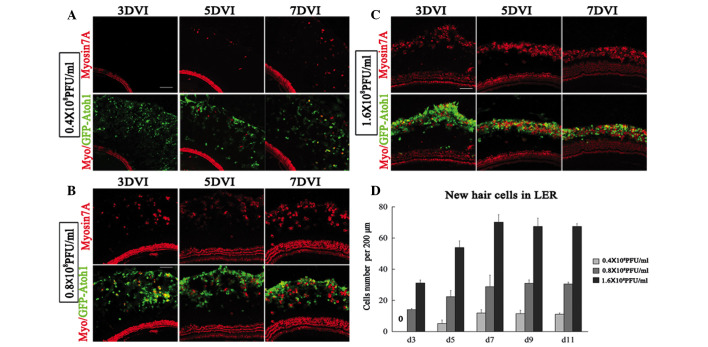
Higher Atoh1 expression levels reduced the duration of EHCLCs formation in the LER. (A) Explants following *Ad-EGFP-atoh1* infection at 0.4×10^8^ PFU/ml from 3–7 DVI; (B) 0.8×10^8^ PFU/ml from 3–7 DVI; (C) 1.6×10^8^ PFU/ml from 3–7 DVI. (D) A bar graph demonstrates that the number of ectopic hair cells increases with time from 7–10 DVI, at different titers [x-axis: days after virus infection (virus titer); y-axis: number of EHCLCs]. Scale bar: 100 μm in (A), (B) and (C). Atoh1, Atonal homolog1; EHCLCs, ectopic hair-cell-like cells; LER, lesser epithelial ridge; Ad5, human adenovirus serotype 5; DVI, days after viral infection.

The number of Atoh1-induced EHCLCs increased with time. When applied to cultured explants with 0.16×10^8^ PFU/ml *Ad5-EGFP-atoh1*, no myosin-positive cells were detected even at 11 DVI. At 0.4×10^8^ PFU/ml *Ad5-EGFP-atoh1*, 5±2 EHCLCs per 200 μm were detected as early as 5 DVI in the LER, which increased to 12±2 at 7 DVI, 11±2 at 9 DVI and 11±1 at 11 DVI (Fig. 4A and D). At 0.8×10^8^ PFU/ml *Ad5-EGFP-atoh1*, 14±1 EHCLCs per 200 μm were detected as early as 3 DVI in the LER, which increased at 22±4 on 5 DVI, 29±7 at 7 DVI, 31±2 at 9 DVI and 31±1 at 11 DVI (Fig. 4B and D). At 1.6×10^8^ PFU/ml *Ad5-EGFP-atoh1*, we detected myosin7A-positive cells as early as 60 h following *atoh1* infection, 31±2 EHCLCs per 200 μm were detected at 3 DVI in the LER, which increased to 54±4 on 5 DVI, 70±5 on 7 DVI, 67±6 on 9 DVI and 67±2 on 11 DVI (Fig. 4C and D). Therefore, at a low titer (0.4×10^8^ PFU/ml) with low Atoh1 expression, EHCLC formation required a longer time (5 days). At a higher titer (1.6×10^8^ PFU/ml) with high Atoh1 expression, however, EHCLC formation required only 2.5 days. EHCLCs increased with time but remained constant from 7–11 days in all groups (Fig. 4D). In conclusion, the Atoh1 expression level critically affected the time required for EHCLC formation.

### Atoh1 expression defines the fate of LER cells

**Figure 5:**
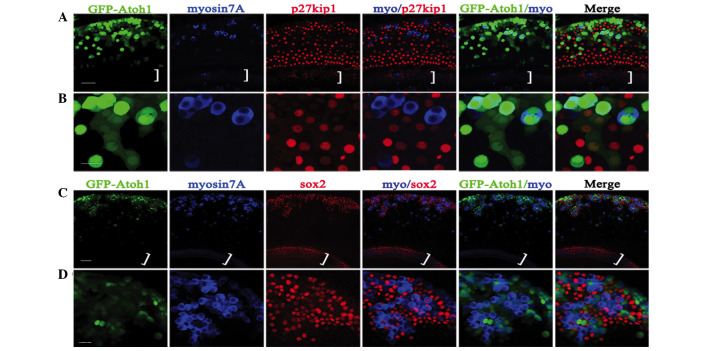
Atoh1 expression defines the fate of LER cells. (A) Explants at 0.8×108 PFU/ml on 3 DVI. A number of GFP-positive LER cells (green) were already myosin7A positive (blue), while others were p27kip1 positive (red). (B) Magnified images from (A). (C) Explants at 0.8×10^8^ PFU/ml at 7 DVI. A number of GFP-positive LER cells (green) were already myosin7A positive (blue), while sox2-positive cells (red) surrounded the EHCLCs. (D) Magnified images from (C). Scale bar: 50 μm in (A) and (C); 20 μm in (B) and (D). Atoh1, Atonal homolog1; EHCLCs, ectopic hair-cell-like cells; LER, lesser epithelial ridge; Ad5, human adenovirus serotype 5; DVI, days after viral infection.

The data indicated that the number of Atoh1-induced EHCLCs increased with time but this effect ceased at 7–11 days, regardless of the titer (Fig. 4D). Despite infection of cultured cochlear explants with *Ad*-*EGFP*-*atoh1* at 1.6×10^8^ PFU/ml, only ~71% of infected cells in the LER were able to transdifferentiate into hair cell-like cells. Following *Ad-EGFP-atoh1* infection, a number of the infected LER cells (EGFP positive) transformed to hair-cell-like cells (myosin7A positive) with an oblong or round shape, whereas other cells remained nonsensory epithelial cells (p27kip1) with a polygonal, flat shape (Fig. 5A and B). Furthermore, many myosin7A-positive cells clustered in the LER with sox2-positive cells surrounding them, indicating that hair-cell-like cells may induce supporting cell formation (Fig. 5C and D).

## Discussion

The human Ad5 vector, encoding both Atoh1 and the reporter gene EGFP, is a useful tool for new hair cell production due to its high transfection efficiency, low level of target tissue damage and ease of control (6–12,14). The viral titration in the present study indicated that appropriate titers induce optimal Atoh1 expression. At titers >2.4×10^8^ PFU/ml, cochlear cultured explants may be severely damaged. At titers <0.16×10^8^ PFU/ml, although weak Atoh1 expression was observed, it was not sufficient to generate ectopic hair cell formation. Our data indicate that 0.4–1.6×10^8^ PFU/ml *Ad5*-*EGFP*-*atoh1* is an efficient and safe titer range for hair-cell-like cell formation in cultured cochlear explants.

At titers of 0.16–1.6×10^8^ PFU/ml, higher infection efficiency and expression levels were observed (Fig. 1). GFP alone did not induce EHCLCs expression, whereas *Ad5*-*EGFP*-*atoh1* did induce robust EHCLCs formation. Thus, EHCLCs formation in the LER was Atoh1 expression dependent. At titers <0.1×10^8^ PFU/ml, although a number of weakly GFP-positive cells were identified, no myosin7A-positive cells were detected, even at 11 DVI. At titers of 0.4×10^8^ PFU/ml *Ad5*-*EGFP*-*atoh1*, only ~4% of *atoh1*-infected cells converted to hair-cell-like cells by 5 DVI in the LER. At titers of 1.6×10^8^ PFU/ml, ~54% of *atoh1*-infected cells converted to hair cell-like cells. These data suggest that hair cell formation requires a certain level of Atoh1, with higher expression inducing more hair cell-like cell formation.

At 0.4–1.6×10^8^ PFU/ml *Ad5*-*EGFP*-*atoh1*, EHCLC formation increased with time. At 0.4×10^8^ PFU/ml, EHCLC formation required 5 days. However, at 1.6×10^8^ PFU/ml, ectopic hair cell formation only required 2.5 days. Thus, greater Atoh1 expression shortens the time required for EHCLC formation. Furthermore, the number of EHCLCs increased with time but then ceased increasing at 7 DVI for all titers. Even at 1.6×10^8^ PFU/ml, only ~71% of *Ad5*-*EGFP*-*atoh1*-infected cells in the LER transdifferentiated into hair cell-like cells. The fate of the non-differentiated cells may help explain this phenomenon.

The data from the present study further revealed that hair cell formation requires a certain Atoh1 expression level; if it was too low, the LER did not convert into hair cell-like cells. A number of *Ad5*-*EGFP*-*atoh1*-infected LER cells (EGFP positive) had already converted to hair cell-like cells (myosin7A positive) with an oblong or round shape at 3 DVI, while the other LER cells remained nonsensory epithelial cells (p27kip1 positive) with a polygonal, flat shape (Fig. 5A and B). At 3 DVI, the majority of myosin7A-positive cells exhibited a strong green fluorescence and p27kip1-positive cells appeared to have weak or no green fluorescence. However, numerous myosin7A-positive cells were observed clustered in the LER with sox2-positive cells surrounding them (Fig. 5C and D), indicating that hair cell-like cells induce supporting cell formation, which has also been previously reported (10). Therefore, the majority of *Ad5*-*EGFP*-*atoh1*-infected LER cells (with sufficient Atoh1 expression) converted into hair cells and induced the surrounding nonsensory epithelial cells to transform into supporting cells.

In the present study, an appropriate virus titer range for infecting cultured cochlear explants was examined, providing highly efficient infection and conversion rates but reducing the infection side effects. Atoh1 expression is critical to hair cell formation, as it defines the fate of LER cells as either hair cell-like cells or nonsensory epithelial cells. The present study provides an important guideline for future investigations to develop novel gene therapy strategies in the treatment of deafness.
